# Supplementary material for: COVID-19 testing protocols to guide duration of isolation: a cost-effectiveness analysis
Source: BMC Public Health. 2023 May 11;23:864. doi: 10.1186/s12889-023-15762-0 (PMC10173903; doi:10.1186/s12889-023-15762-0)
Supplement: Supplementary file 1 — Supplementary Material 1 [file 12889_2023_15762_MOESM1_ESM.docx]

**Supplement to: Cost-effectiveness of antigen testing for ending COVID-19 isolation**

**Calculation of “residual R”**

An adjusted secondary reproduction number (R_eff_) was used in the model to determine the number of secondary infections transmitted by each individual leaving isolation. Since the majority of infections would have occurred prior to infection, and the time when which patients would spread most virus were spent in isolation, we calculated a “residual R” for base-case and scenario analyses. We assumed that the portion of secondary transmissions on each day would be proportional to viability of SARS-CoV-2 over time. Based on findings from Wolfel et al.[1] describing proportion of severe COVID-19 patient samples containing viable virus, we defined a distribution for the portion of R_eff_ transmitted on each day. We then applied this distribution to the total R_eff_ value to determine secondary infections transmitted on each day (Table S1). Other studies[2-5] of samples from asymptomatic and/or mild COVID-19 patients described similar patterns of infectivity as Wolfel et al., but they lacked quantitative data points that could be applied to our model. Given the similarities in description, we concluded Wolfel et al. provided a reasonable proxy.

**Table S1.** Distribution of R_eff_ across the infectious period.

| Day since COVID-19 confirmation | Percent of samples with viable virus | Portion of R-eff transmitted | Number of infections transmitted |
| --- | --- | --- | --- |
| 0 (day of confirmation) | 100% | 13% | 0.16 |
| 1 | 100% | 13% | 0.16 |
| 2 | 100% | 13% | 0.16 |
| 3 | 100% | 13% | 0.16 |
| 4 | 100% | 13% | 0.16 |
| 5 | 90% | 12% | 0.14 |
| 6 | 70% | 9% | 0.11 |
| 7 | 50% | 7% | 0.08 |
| 8 | 30% | 4% | 0.05 |
| 9 | 10% | 1% | 0.02 |
| 10 | 0% | 0% | 0 |
| Total |  | 100% | 1.2 |

**Calculation of COVID-19 medical costs**

We calculated a weighed average cost of COVID-19 treatment by disease severity[6, 7], as outlined in Table S2.

**Table S2.** COVID-19 medical costs.

| **Type of care needed** | **% of infected** | **Cost** |
| --- | --- | --- |
| Does not seek care | 40% | $0 |
| Seeks outpatient care | 36% | $513 |
| Seeks care at ED | 15% | $815 |
| Requires hospitalization | 9% | $12,000 |
| Requires ICU admission | 0.1% | $61,000 |
| **Weighted total** | **100%** | **$1,436** |

**Calculation of R_eff_ for scenario analyses**

For scenario analyses, we defined COVID-19 transmission risk based on the number of contacts, mask adherence, and vaccination rate of contacts following de-isolation. The low-risk scenario was defined as joining the household where everyone is vaccinated and continue to wear masks. The medium-risk scenario was defined as having up to 10 contacts, all fully vaccinated but inconsistent mask wearing. The high-risk scenario was defined as congregate setting or work with more than 10 others with inconsistent vaccination and mask wearing. These factors increased or decreased the risk of transmission at base-case, and these relative risks were used to adjust the base-case R_eff_ of 1.2 for each scenario. The new R_eff_ was then used to calculate the residual R for each scenario with the same approach as in base-case (Table S3).

*Number of contacts:* On average, people in the United States had an average of four non-household contacts per day.[8] We assumed a 50% increase in this value to account for changing contact patterns after vaccines were introduced and several states lifted mask mandates, and added an average of three household members, resulting in nine total contacts in base-case.

*Vaccine protection:* Base-case vaccine protection was calculated as 26.5%. This accounted for 86% seroprevalence in the US (either from vaccination or natural immunity)[9], 85% immunity from vaccinations or prior infections during the Delta wave[10], and 64% reduction in protection against Omicron[11].

*Mask wearing:* Mask adherence in the US was 40% as of December 2021.[12] This was adjusted by the 0.38 odds ratio of COVID-19 with masks[13], resulting in 25% protection from mask wearing in base-case.

**Table S3.** Relative risk for SARS-CoV-2 transmission in different risk scenarios.

|  | Base-case | Low-risk | | Medium-risk | | High-risk | |
| --- | --- | --- | --- | --- | --- | --- | --- |
|  | Value | Value | Relative risk | Value | Relative risk | Value | Relative risk |
| Number of contacts | 9 | 3 | 0.33 | 10 | 1.11 | 20 | 2.22 |
| Vaccine protection | 26.5% | 30.9% | 0.84 | 30.9% | 0.84 | 15.4%** | 1.42 |
| Mask wearing | 24.8% | 24.0%* | 1.03 | 24.8% | 1.00 | 24.8% | 1.00 |
| Cumulative relative risk on R_eff_ |  |  | 0.29 |  | 0.93 |  | 3.15 |
| R_eff_ | 1.2 | 0.35 |  | 1.11 |  | 3.78 |  |
| Residual R | 0.26 | 0.07 |  | 0.24 |  | 0.81 |  |

*Even though everyone wore masks at home, we doubled the odds ratio for COVID-19 to account for household contacts spending more time together in closer proximity.

**Assuming 50% are vaccinated.

**References**

1. Wolfel R, Corman VM, Guggemos W, Seilmaier M, Zange S, Muller MA, Niemeyer D, Jones TC, Vollmar P, Rothe C *et al*: **Virological assessment of hospitalized patients with COVID-2019**. *Nature* 2020, **581**(7809):465-469.

2. Walsh KA, Jordan K, Clyne B, Rohde D, Drummond L, Byrne P, Ahern S, Carty PG, O'Brien KK, O'Murchu E *et al*: **SARS-CoV-2 detection, viral load and infectivity over the course of an infection**. *J Infect* 2020, **81**(3):357-371.

3. He X, Lau EHY, Wu P, Deng X, Wang J, Hao X, Lau YC, Wong JY, Guan Y, Tan X *et al*: **Temporal dynamics in viral shedding and transmissibility of COVID-19**. *Nat Med* 2020, **26**(5):672-675.

4. Perera RAPM, Tso E, Tsang OTY, Tsang DNC, Fung K, Leung YWY, Chin AWH, Chu DKW, Cheng SMS, Poon LLM *et al*: **SARS-CoV-2 Virus Culture and Subgenomic RNA for Respiratory Specimens from Patients with Mild Coronavirus Disease**. *Emerg Infect Dis* 2020, **26**(11):2701-2704.

5. Singanayagam A, Patel M, Charlett A, Lopez Bernal J, Saliba V, Ellis J, Ladhani S, Zambon M, Gopal R: **Duration of infectiousness and correlation with RT-PCR cycle threshold values in cases of COVID-19, England, January to May 2020**. *Euro Surveill* 2020, **25**(32).

6. Yek C, Warner S, Wiltz JL, Sun J, Adjei S, Mancera A, Silk BJ, Gundlapalli AV, Harris AM, Boehmer TK *et al*: **Risk Factors for Severe COVID-19 Outcomes Among Persons Aged ≥18 Years Who Completed a Primary COVID-19 Vaccination Series — 465 Health Care Facilities, United States, December 2020–October 2021**. *MMWR* 2022, **71**(1):19-25.

7. **Potential costs of COVID-19 treatment for people with employer coverage** [<https://www.healthsystemtracker.org/brief/potential-costs-of-coronavirus-treatment-for-people-with-employer-coverage/>]

8. Feehan DM, Mahmud AS: **Quantifying population contact patterns in the United States during the COVID-19 pandemic**. *Nat Commun* 2021, **12**(1):893.

9. **COVID-19 Seroprevalence Data** [<https://www.cdph.ca.gov/Programs/CID/DCDC/Pages/COVID-19/Sero-prevalence-COVID-19-Data.aspx>]

10. Hall VJ, Foulkes S, Charlett A, Atti A, Monk EJM, Simmons R, Wellington E, Cole MJ, Saei A, Oguti B *et al*: **SARS-CoV-2 infection rates of antibody-positive compared with antibody-negative health-care workers in England: a large, multicentre, prospective cohort study (SIREN)**. *The Lancet* 2021, **397**(10283):1459-1469.

11. Yang W, Shaman J: **SARS-CoV-2 transmission dynamics in South Africa and epidemiological characteristics of the Omicron variant**. *medRxiv* 2021:2021.2012.2019.21268073.

12. **COVID-19 Projections** [<https://covid19.healthdata.org/united-states-of-america?view=mask-use&tab=trend>]

13. Li Y, Liang M, Gao L, Ayaz Ahmed M, Uy JP, Cheng C, Zhou Q, Sun C: **Face masks to prevent transmission of COVID-19: A systematic review and meta-analysis**. *Am J Infect Control* 2021, **49**(7):900-906.
